# Author Correction: Rapid *de novo* assembly of the European eel genome from nanopore sequencing reads

**DOI:** 10.1038/s41598-019-44275-3

**Published:** 2019-05-22

**Authors:** Hans J. Jansen, Michael Liem, Susanne A. Jong-Raadsen, Sylvie Dufour, Finn-Arne Weltzien, William Swinkels, Alex Koelewijn, Arjan P. Palstra, Bernd Pelster, Herman P. Spaink, Guido E. van den Thillart, Ron P. Dirks, Christiaan V. Henkel

**Affiliations:** 1 ZF-screens B.V., Leiden, The Netherlands; 2 Institute of Biology, Leiden University, Leiden, The Netherlands; 3 Muséum National d’Histoire Naturelle, Sorbonne Universités, Research Unit BOREA, Biology of Aquatic Organisms and Ecosystems, CNRS, IRD, UCN, UA, Paris, France; 4 Norwegian University of Life Sciences, Faculty of Veterinary Medicine, Department of Basic Science and Aquatic Medicine, Oslo, Norway; 5 DUPAN Foundation, Wageningen, The Netherlands; 6 Animal Breeding and Genomics Centre, Wageningen Livestock Research, Wageningen University & Research, Wageningen, The Netherlands; 7 Institute of Zoology and Center for Molecular Biosciences, University of Innsbruck, Innsbruck, Austria; 8 University of Applied Sciences Leiden, Leiden, The Netherlands; 9 Generade Centre of Expertise in Genomics, Leiden, The Netherlands

Correction to: *Scientific Reports* 10.1038/s41598-017-07650-6, published online 03 August 2017

This Article contains errors.

Since the publication of this Article, the website hosting the assembly data has become inactive. The data has now been re-deposited in the DataverseNO repository. As such, the corrected Data Availability section should be as follows.

Data Availability

The nanopore sequencing data are available in the European Nucleotide Archive (accession number PRJEB20018). The Racon- and Pilon-corrected candidate assembly is available at 10.18710/NMTGUN. The TULIP-scripts are available at https://github.com/Generade-nl.

