# Supplementary material for: Rapid de novo assembly of the European eel genome from nanopore sequencing reads
Source: Sci Rep. 2017 Aug 3;7:7213. doi: 10.1038/s41598-017-07650-6 (PMC5543108; doi:10.1038/s41598-017-07650-6)
Supplement: Supplementary file 1 — Supplementary Information [file 41598_2017_7650_MOESM1_ESM.pdf]

Supplementary Information for
Rapid *de novo* assembly of the European eel genome from nanopore sequencing reads

Supplementary Table S1: Nanopore sequencing 2

Supplementary Table S2: *A. anguilla* genome assemblies using TULIP 2

Supplementary Table S3: Characteristics of the *A. anguilla* candidate assembly 3

Supplementary Figure S1: GenomeScope *k*-mer profiles 4

Supplementary Figure S2: Misassembly scenarios 5

Supplementary Figures S3–S7: Local graph neighborhoods of scaffold inconsistencies 6–11

Supplementary Table S1. Nanopore sequencing

Chemistry	Total yield	Read N50	Longest read
R7.3 2D	245.0 Mbp	10345 bp	71212 bp
R9 1D	4.488 Gbp	19052 bp	233352 bp
R9 2D	975.7 Mbp	8073 bp	45931 bp
R9.4 1D	9.920 Gbp	11852 bp	215759 bp

Supplementary Table S2. A. anguilla *genome assemblies using TULIP*

Seed size	Seed number	Read selection	Scaffold N50*	Nr. of scaffolds	Assembly size*
285 bp	*873k*	*100%*	*1170852 bp*	*2366*	*849.7 Mbp*
285 bp	873k	75%	697683 bp	3531	839.0 Mbp
285 bp	873k	50%	341223 bp	6919	815.0 Mbp
285 bp	873k	25%	90534 bp	21764	730.4 Mbp
285 bp	437k	100%	719956 bp	3173	802.6 Mbp
285 bp	218k	100%	361910 bp	4889	709.6 Mbp
270 bp	1746k	100%	1185122 bp	2805	875.6 Mbp
270 bp	1310k	100%	1300479 bp	2317	866.7 Mbp
270 bp	873k	100%	1176872 bp	2330	851.0 Mbp
270 bp	437k	100%	711245 bp	3132	802.6 Mbp

* *Sizes based on mean distances between seeds.*

Supplementary Table S3. Characteristics of the A. anguilla *candidate assembly*

**Statistic**	**Value**	**Note**
Number of scaffolds	2366	
Seed graph scaffold N50	1.17 Mbp	cf. Table 4
Seed graph assembly sum	849.7 Mbp	cf. Table 4
Uncorrected scaffold N50	1.19 Mbp	
Uncorrected scaffold sum	863.3 Mbp	
Racon scaffold N50	1.21 Mbp	
Racon assembly sum	881.3 Mbp	
Pilon scaffold N50	1.23 Mbp	
Pilon assembly sum	891.7 Mbp	
Alignment time	7 hours	1 thread*
Seed graph time	51 minutes	1 thread
Sequence addition time	14 minutes	1 thread
Racon correction time	~22 hours	1 thread*
Pilon correction time	~24 hours	1 thread*

* *These stages can be sped up by multithreading. For example, the actual alignment was run with four concurrent threads in 2 hours, 34 minutes.*

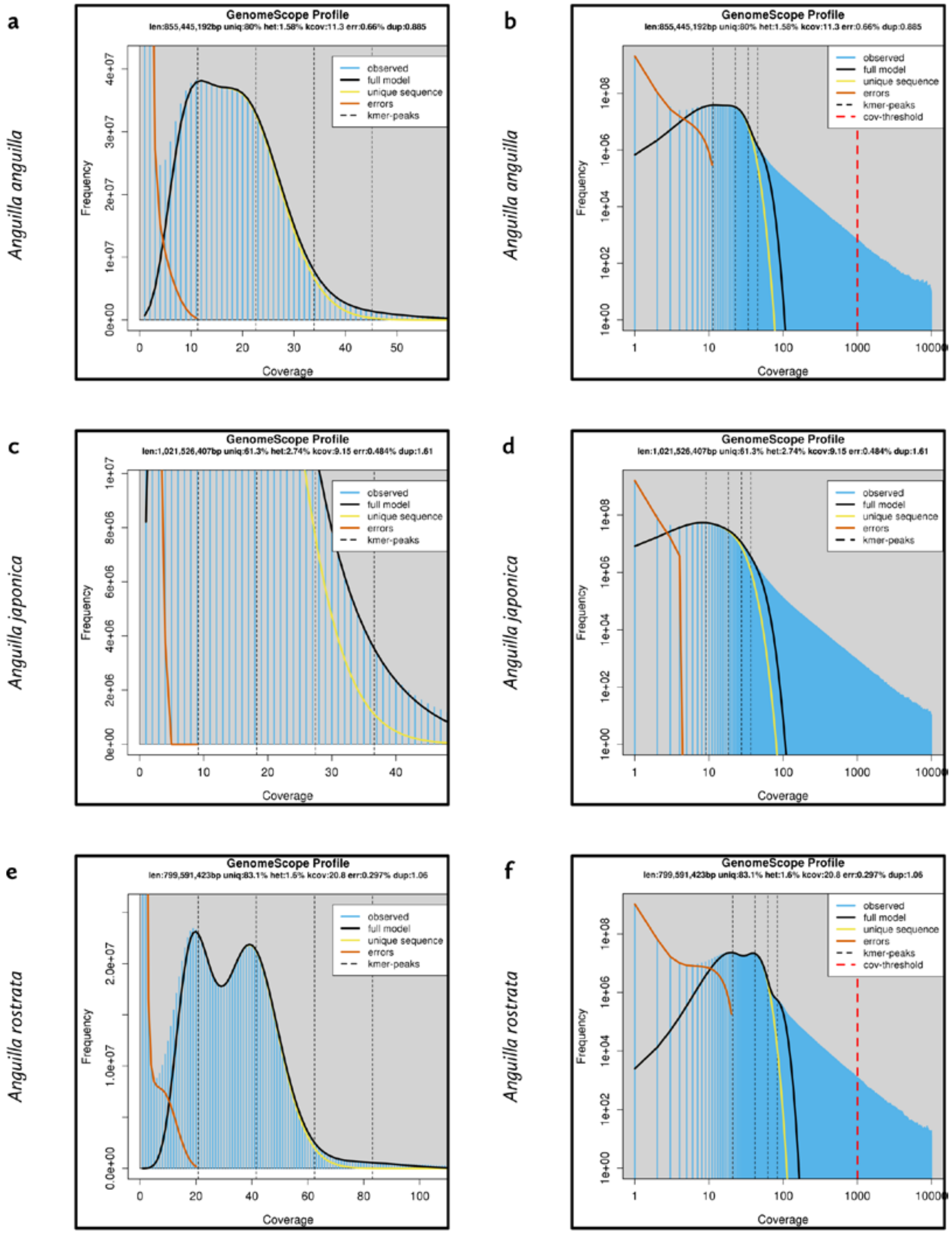

Supplementary Figure S1. GenomeScope k*-mer profiles*

Shown are the 19-mer profile analyses for **(a)** *A. anguilla*, **(b)** *A. japonica* and **(c)** *A. rostrata*. Both regular and logarithmic scale plots are included. The full analyses are available at the GenomeScope website (*http://qb.cshl.edu/genomescope/analysis.php*) using the codes TDVyqzdJXugs2lEcd2AB (*A. anguilla*), VtNZvSlV7nzfq6yvTlAp (*A. japonica*) and 8citu1cxv9SHXOzqbA43 (*A. rostrata*).

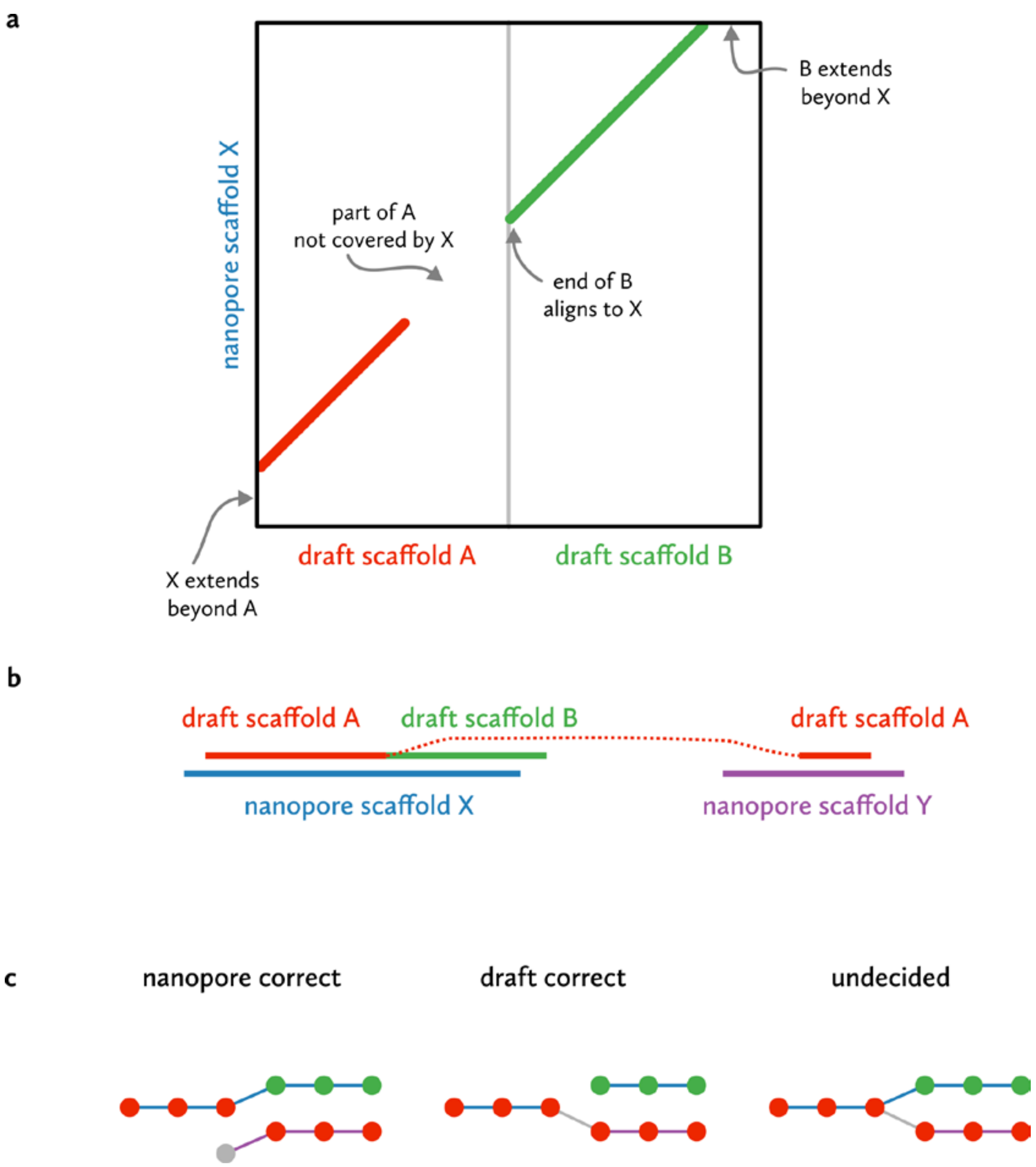

Supplementary Figure S2. Misassembly scenarios

If draft scaffolds do not align completely to a single nanopore scaffold, this is apparent in the alignment plot **(a)**. The origins of the actual situation **(b)** can be gleaned from the nanopore graph **(c)**. Based on the local graph context around the inconsistency, multiple explanations are possible: nanopore evidence can exist to support the nanopore scaffolds only (in which case the draft scaffold is probably incorrect), to support the draft scaffold only (in which case the nanopore scaffold is incorrect), or to support both (in which case additional evidence needs to be examined to determine the correct scaffolding path).

Supplementary Figures S3–S7. Local graph neighborhoods of scaffold inconsistencies

For each of the inconsistencies identified in Fig. 5b–f, the local neighborhood in the initial seed graph is shown (similar to Fig. 3 and Supplementary Fig. 2c). Red and green nodes represent seeds that align to the truncated old scaffold and its non-truncated neighbor, respectively. Grey nodes do not align to these scaffolds (or at least, not locally), yellow nodes align partially to two scaffolds. The final extracted TULIP scaffold paths are indicated by blue arrows. As in the draft the 'red' scaffolds do not end at the joins to the 'green' scaffolds, an alternative path possibility of continuing with 'red' seeds would be expected at this point. In none of the cases examined does this appear to be the case.

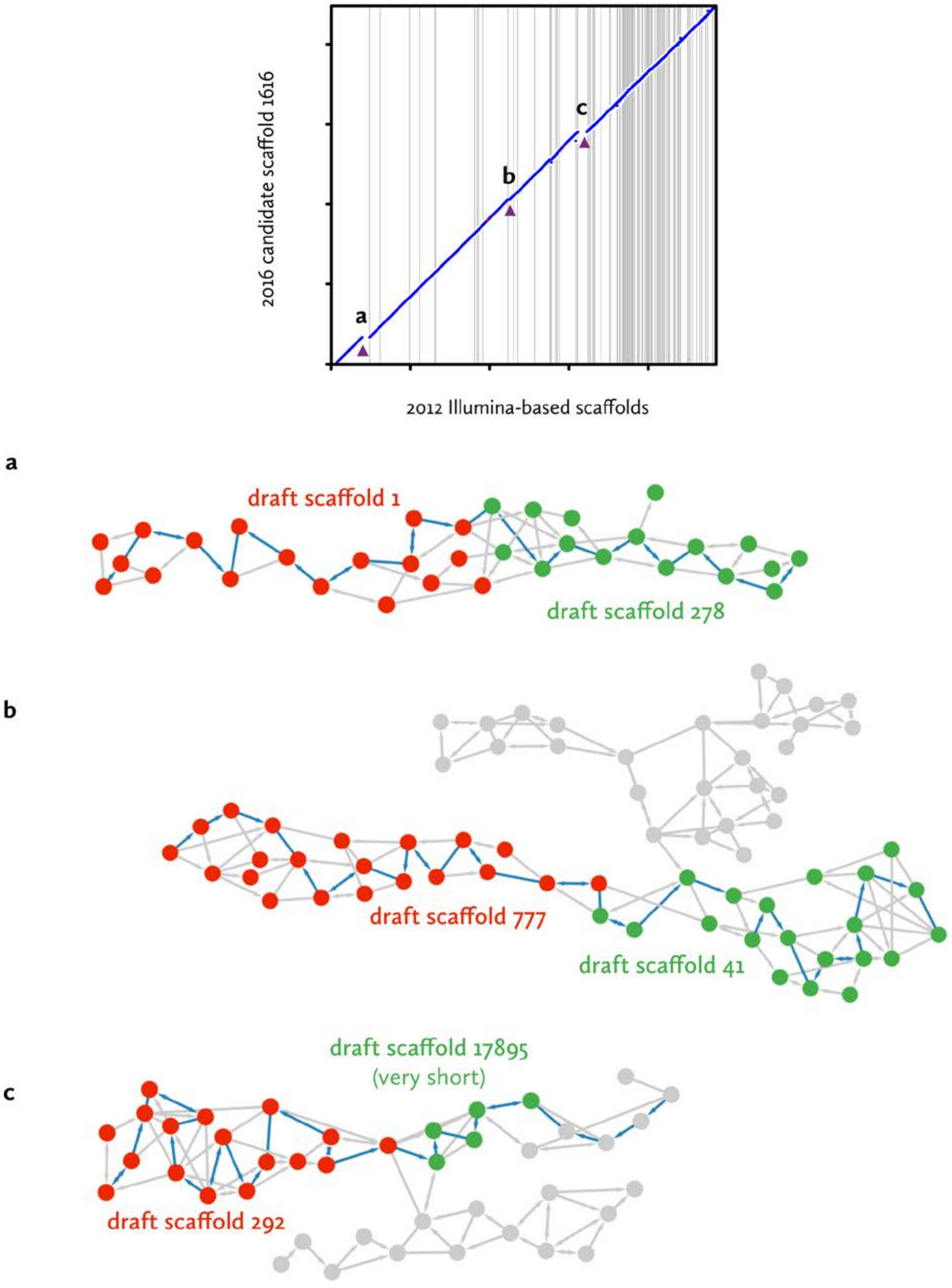

Supplementary Figure S3

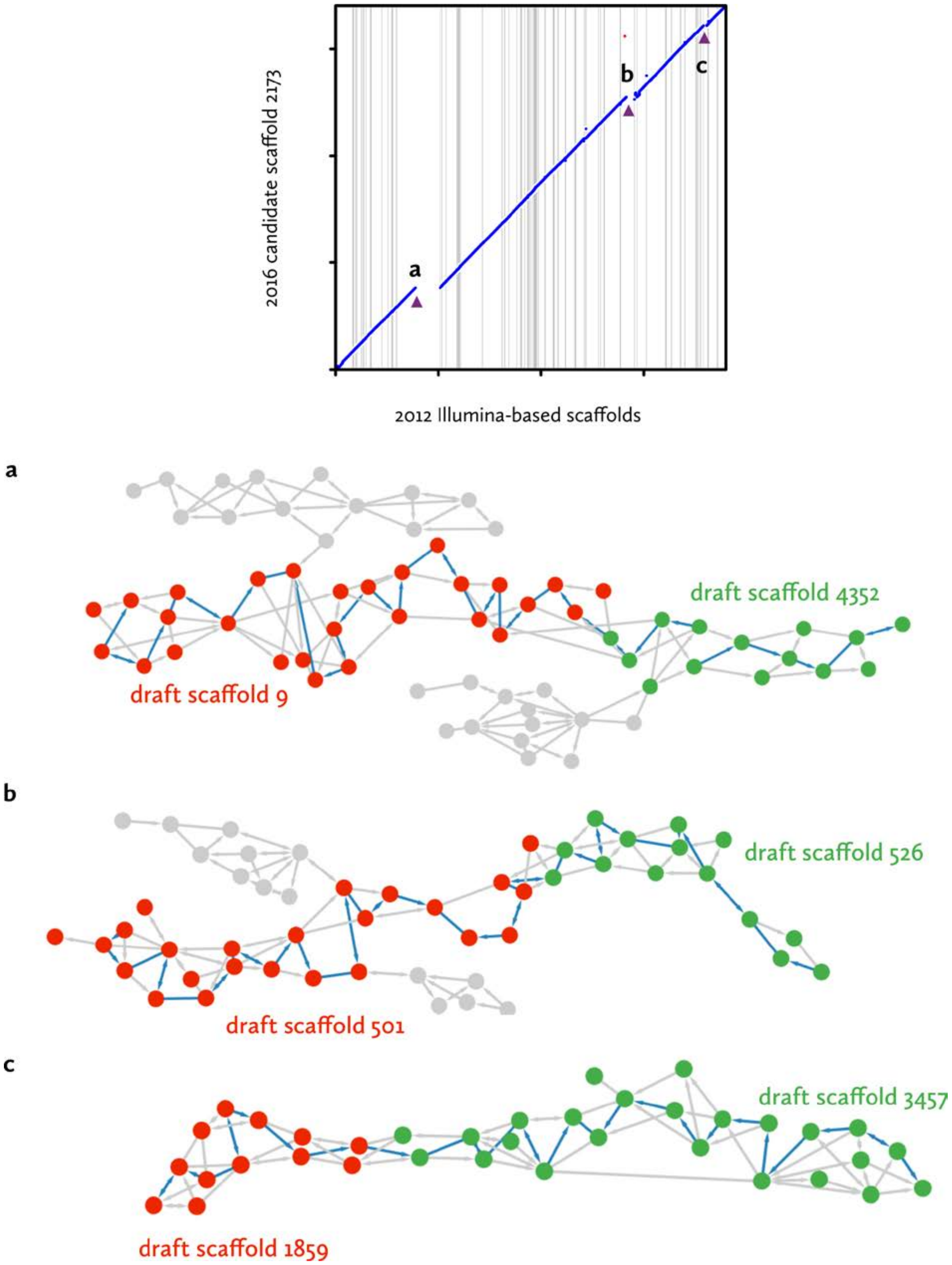

Supplementary Figure S4

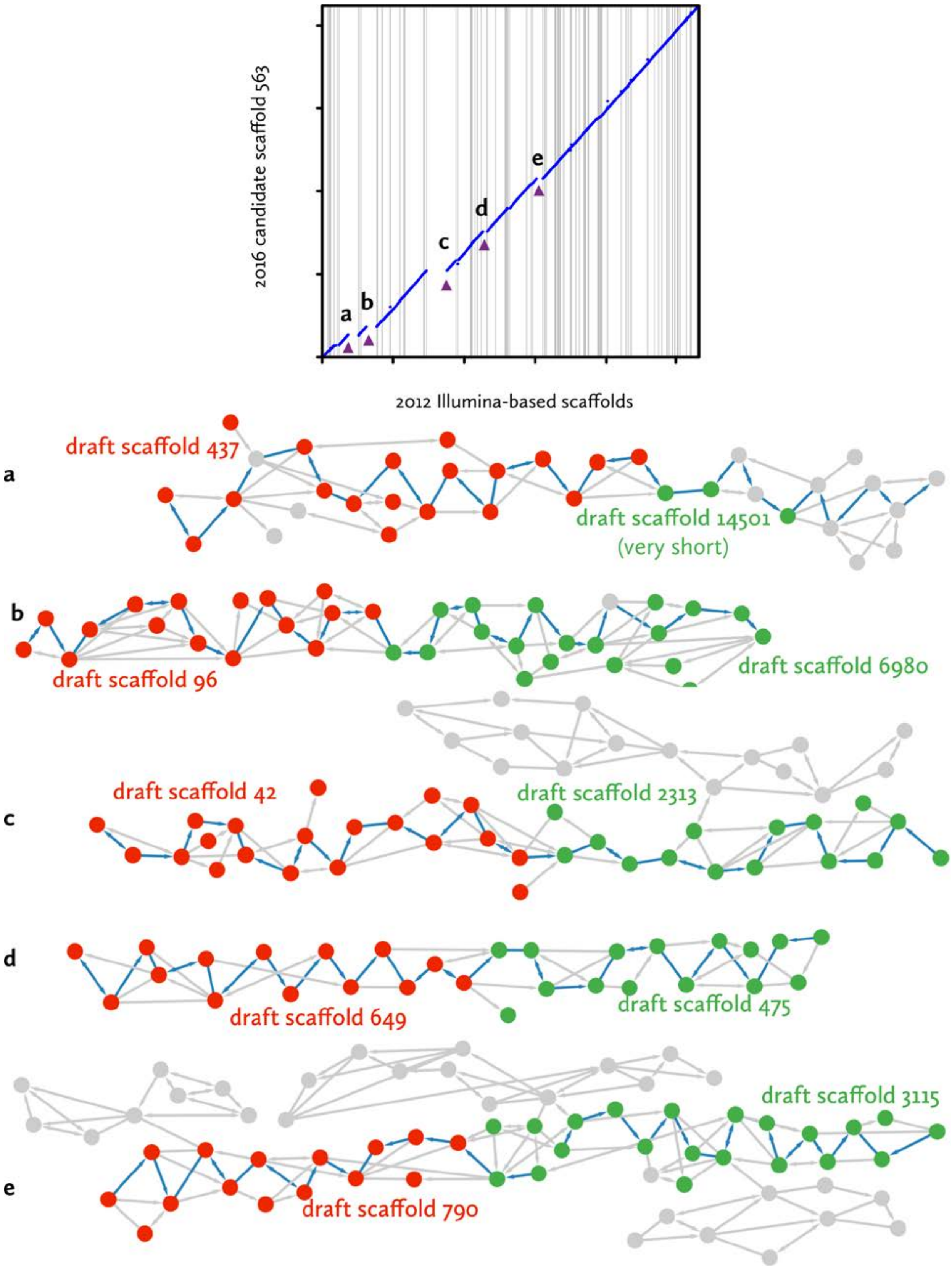

Supplementary Figure S5

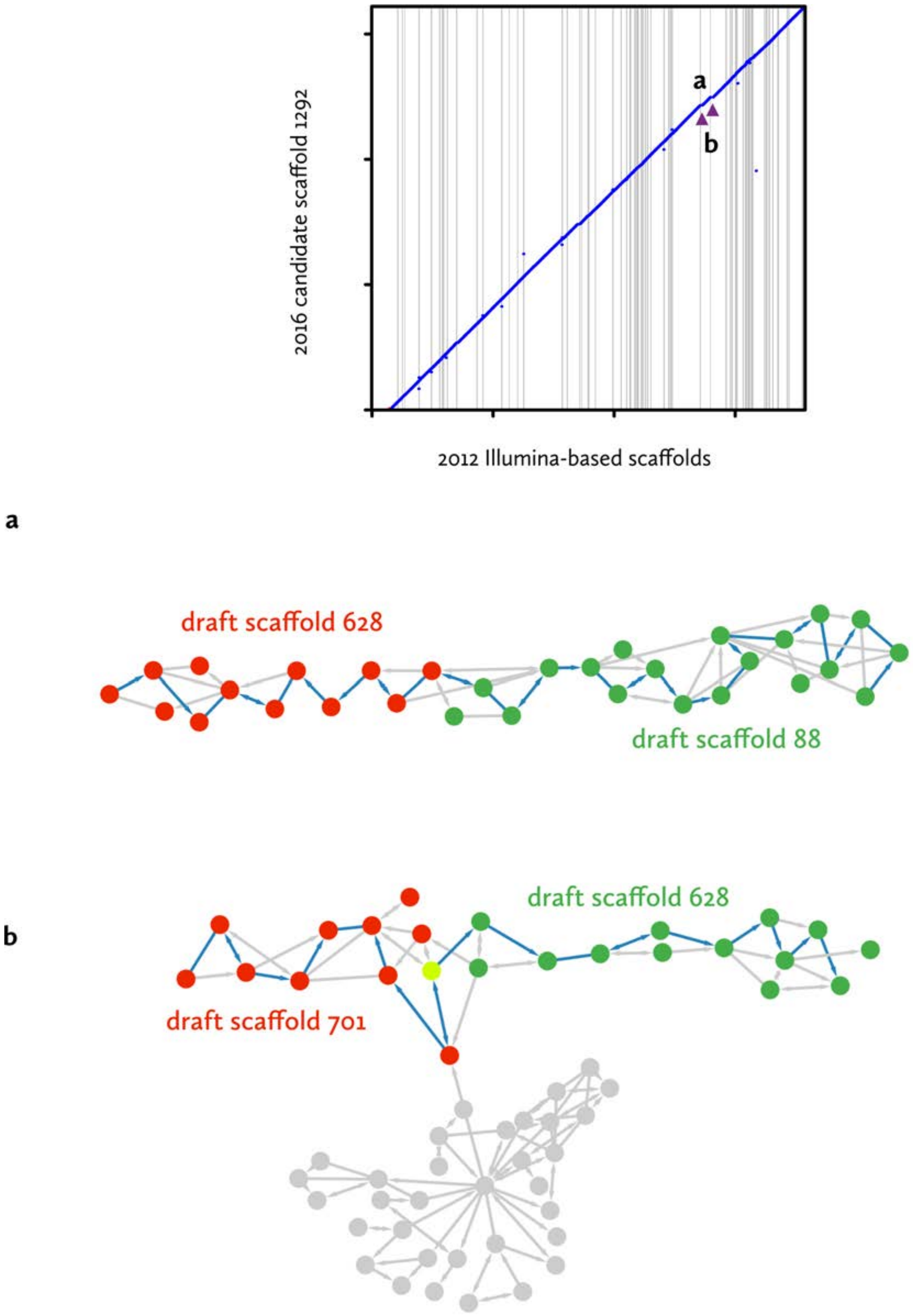

Supplementary Figure S6

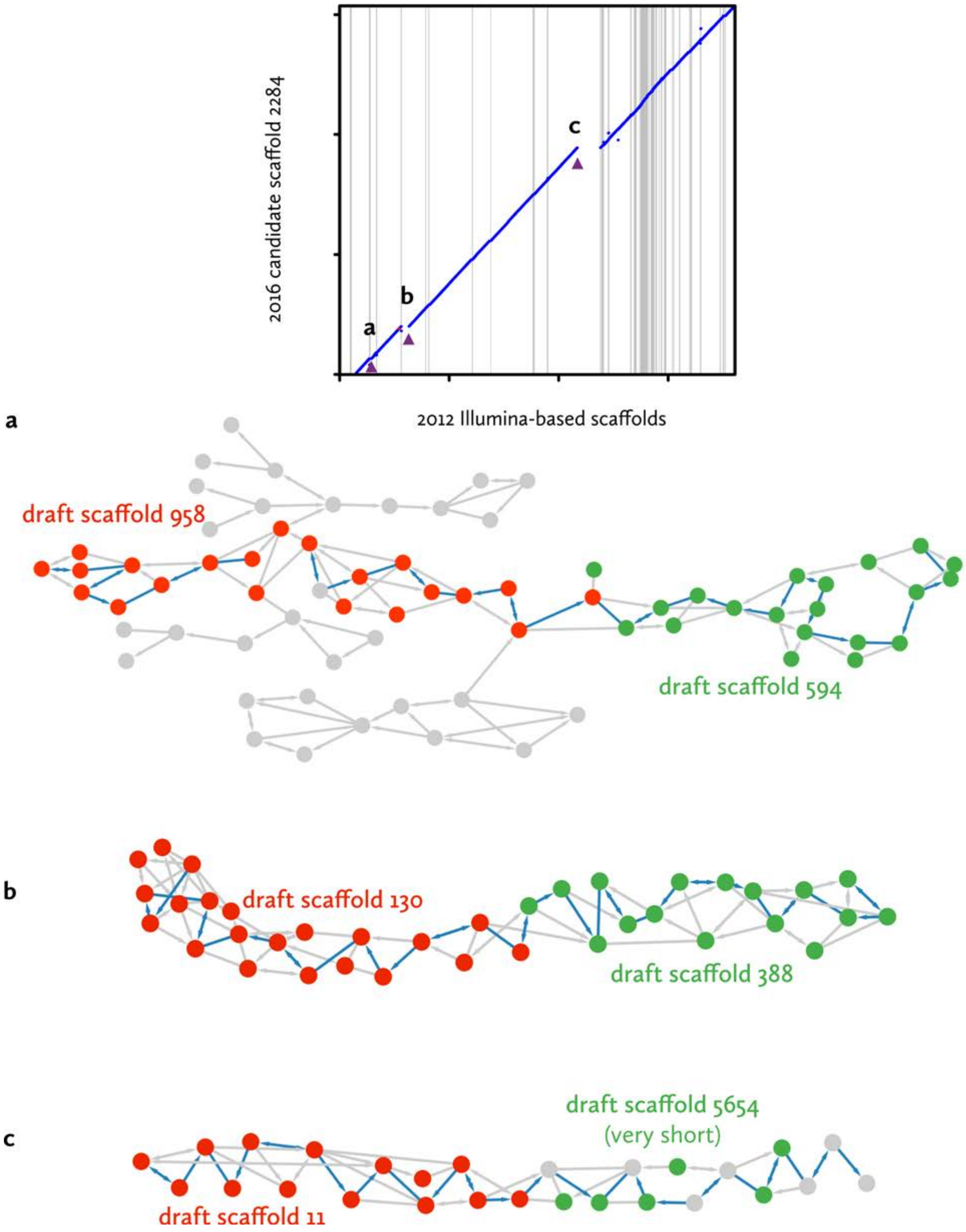

Supplementary Figure S7
